# Pregnancy outcomes and mother-to-child transmission rate in HTLV-1/2 infected women attending two public hospitals in the metropolitan area of Rio de Janeiro

**DOI:** 10.1371/journal.pntd.0007404

**Published:** 2019-06-10

**Authors:** Danielle Bittencourt Sodré Barmpas, Denise Leite Maia Monteiro, Stella Regina Taquette, Nádia Cristina Pinheiro Rodrigues, Alexandre José Baptista Trajano, Juliana de Castro Cunha, Camila Lattanzi Nunes, Lucia Helena Cavalheiro Villela, Sérgio A. M. Teixeira, Denise Cardoso das Neves Sztajnbok, Márcio Neves Bóia

**Affiliations:** 1 Department of Obstetrics, Universidade do Estado do Rio de Janeiro (UERJ), Rio de Janeiro, RJ, Brazil; 2 Department of Gynecology and Obstetrics, Centro Universitário Serra dos Órgãos (UNIFESO), Teresópolis, RJ, Brazil; 3 Department of Internal Medicine, Universidade do Estado do Rio de Janeiro (UERJ), Rio de Janeiro, RJ, Brazil; 4 Department of Epidemiology, Universidade do Estado do Rio de Janeiro (UERJ), Rio de Janeiro, RJ, Brazil; 5 Department of Epidemiology, Escola Nacional de Saúde Pública (Fiocruz), Rio de Janeiro, RJ, Brazil; 6 Department of Gynecology and Obstetrics, Universidade Unigranrio, Rio de Janeiro, RJ, Brazil; 7 Laboratory of Clinical Pathology, Universidade do Estado do Rio de Janeiro (UERJ), Rio de Janeiro, RJ, Brazil; 8 Department of Pediatrics, Universidade do Estado do Rio de Janeiro (UERJ), Rio de Janeiro, RJ, Brazil; 9 Department of Infectology, Universidade do Estado do Rio de Janeiro (UERJ), Rio de Janeiro, RJ, Brazil; Hospital Universitário Professor Edgard Santos, BRAZIL

## Abstract

HTLV-1/2 are transmitted sexually, by whole cell blood products and from mother-to-child (MTC), mainly through breastfeeding. HTLV-1/2 prevalence in pregnant women is high in Rio de Janeiro, however there were no local studies addressing the rate of adverse pregnancy outcomes (APO) and MTC transmission. The aim was to study sociodemographic characteristics which may be associated to HTLV-1/2 infection and describe pregnancy outcomes and MTC transmission in HTLV-1/2-positive women. The cross-sectional study screened 1,628 pregnant women in of Rio de Janeiro (2012–2014) and found 12 asymptomatic carrier mothers (prevalence = 0.74%). Pregnancy outcome information was retrieved from medical records. Sociodemographic characteristics were similar between the positive and negative groups except for maternal age, which was higher in carrier mothers. The incidence of adverse pregnancy outcomes was similar in infected and non-infected patients (p = 0.33), however there was a high rate of premature rupture of membranes (PROM) amid infected mothers (3/12). Multilevel logistic regression found that for each additional year of age, the chance of being HTLV-1/2-positive increased 11% and that having another sexually transmitted infection (STI) increased 9 times the chance of being infected. Carrier mothers had more antenatal visits (OR = 5.26). Among the children of HTLV-1/2-positive mothers there was one fetal death, one infant death and one loss of follow-up. After two years of follow-up there was one case of MTC transmission (1/9). The mother reported breastfeeding for one month only. Knowledge about factors associated to HTLV-1/2 infection, its impact on pregnancy outcomes and the MTC transmission rate is important to guide public health policies on antenatal screening and management.

## Author summary

HTLV-1/2 are retroviruses transmitted by sex, blood products and from mother to child (MTC), mainly through breastfeeding. There is currently no vaccine, treatment or cure. Although it’s mostly asymptomatic it can cause disabling and even lethal diseases in carriers. The prevalence of HTLV-1/2 in pregnant women at the metropolitan area of Rio de Janeiro is high (0.74%). Our aim was to study the sociodemographic characteristics which may be associated to HTLV-1/2 infection and describe pregnancy outcomes and MTC transmission in the infected population. Apart from being slightly older, there were no differences in the carrier mothers’ sociodemographic profile. Pregnant women with sexually transmitted infections had a 9-fold chance of being HTLV-1/2 positive. Although adverse pregnancy outcomes were not increased, infected mothers had a high rate of ruptured membranes. Among the children of HTLV-1/2-positive mothers there was one fetal death, one infant death and one loss of follow-up. There was one case of MTC transmission (1/9), after one month of breastfeeding. Knowledge about factors associated to HTLV-1/2 infection, its impact on pregnancy, and the MTC transmission rate is important to guide further research and public health policies.

## Introduction

Human T-lymphotropic virus types 1 and 2 (HTLV-1/2) are human oncogenic retroviruses first identified in the early 1980’s [1]. There are six subtypes of HTLV-1 (A to F), which have no impact on the clinical expression of the disease [2]. There are two other types of HTLV (3 and 4); but there is no evidence of their pathogenicity in humans [3].

HTLV-1/2 viruses are globally distributed and there may be up to 10 million infected worldwide [4]. Prevalence is characterized by endemic clusters occurring next to low prevalence areas. It also varies considerably according to the ethnical and social background of the population. Since transmission occurs through infected body fluids, intravenous (IV) drug users and sex workers have been reported as high-risk groups [4]. The association between low social and economic level and lower education is not homogeneous among studies and most likely represents a bias. Endemic HTLV-1 clusters are found in Sub-Saharan Africa, South-western Japan, Central and South America as well as the Middle East and Melanesia [4]. Regardless of the area, seroprevalence increases with age, particularly in women due the excess efficiency of the male-female sexual transmission. HTLV-2 is endemic in Pygmy tribes of Central Africa and in several Native American populations, particularly in the Amazon area [5,6]. It is also frequent in IV drug users, often in co-infection with HIV [5,7].

Brazil may be the country with the highest absolute number of HTLV-1/2 carriers in the world. Estimates range from 800,000 to 2.5 million people [5,8,9]. Such variation in numbers can be explained both by the epidemiological characteristic of the infection and by the lack of data, with large areas of the country unmapped.

Infection is perennial and most of the patients are asymptomatic reservoirs, sustaining the chain of transmission. In contrast, up to 8% of HTLV-1 carriers develop severe diseases, mainly the highly aggressive adult T-cell leukaemia/lymphoma (ATLL) and the painful and disabling HTLV-1-associated myelopathy/Tropical Spastic Paraparesis. Type 1 virus also causes a spectrum of inflammatory conditions, such as dermatitis and uveitis [10]. In turn, HTLV-2 has been associated to erythrodermatitis, neurologic disorders and opportunistic infections [7].

Literature about the effect of HTLV-1/2 infection on pregnancy outcomes is scarce. Only one study on the subject was published. It was conducted in Africa, between 1986 and 1988, involving 45 HTLV-1/2 positive pregnant women and 90 negative ones. No statistically significant differences were found between the groups regarding neither sociodemographic profile, pregnancy and neonatal outcomes [11].

Only four Brazilian researches on HTLV-1/2 in pregnant women and puerperae assessed previous obstetric history and pregnancy outcome, and they reported only on miscarriage [12–15]. Large regional studies which altogether included over 130,000 pregnant women report miscarriage rates between 22% and 30%, notably higher than that observed in non-infected patients [13–15]. On the other hand, the only research conducted on an endemic area found a pregnancy loss rate of 10%, similar to the general population [12]. Dal Fabbro’s study was the only to report the frequency of two or more previous miscarriages, which was 0.8% in HTLV-1/2 infected women. This number is equivalent to the incidence of recurrent pregnancy losses in the general population [13].

HTLV-1/2 is transmitted via whole cell containing body fluids, mainly through sexual contact, exposure to blood products or *viscera* and from mother-to-child (MTC). The relative importance of each mode of transmission is still largely unknown and most likely it varies with the population involved. In endemic areas such as Japan MTC transmission has been described as the main source of transmission, mainly through breastfeeding [12,16]. Only 2.5–5.0% of children are seroconverted in the absence of breastfeeding while up to 25% are infected if breastfed for over 12 months [16–19]. In fact, a recent Brazilian study found a vertical transmission rate of 50% in children who were breastfed for over 24 months [17]. This research also detected an increased risk of infection in siblings, confirming the trend for familial clustering of the disease [17]. Higher proviral load and antibody titers in maternal blood and breastmilk are also associated with increased MTC transmission rate [17,20–22]. On the other hand, peripartum transmission has been shown to have little impact on the burden of disease [19,23].

The aim of the research was to describe the epidemiological profile of pregnant women diagnosed with HTLV-1/2 in the metropolitan area of Rio de Janeiro, the occurrence of adverse pregnancy outcomes (APO) and the rate of mother-to-child transmission.

## Methods

### Study population

The study population consisted of 1,628 pregnant women. The first 1,204 were enrolled at admission for delivery as part of a research on HTLV-1/2 prevalence conducted at two public hospitals in the metropolitan area of Rio de Janeiro: the ‘Pedro Ernesto’ University Hospital of the Rio de Janeiro State University (Universidade do Estado do Rio de Janeiro–HUPE/UERJ) and the ‘Hospital Estadual da Mãe’ (HEM). HUPE is a referral centre for high-risk patients while HEM, situated at the adjacent city of Mesquita, assists low and medium-complexity cases [24]. As a result of the relevant prevalence found in the local population (0.66%) at the first part of the study, routine HTLV-1/2 screening was instituted in HUPE’s perinatal unit in July 2013. The other 424 women were recruited at their first antenatal visit. During the first part of the study, roughly 24% of the women attending the HEM were recruited while the uptake at UERJ was 40%. During the antenatal screening period at UERJ only four approached women refused to take part in the study. The study was conducted between November 2012 and December 2017. Subjects were recruited until 2014 and the children of HTLV-1/2 positive women were followed for three years. Women who were mentally unable to give consent or who declined to take part in the research were excluded. A structured questionnaire with socio-epidemiological, clinical and reproductive data was applied at recruitment. Women found to be HTLV-1/2 positive were counseled by a multidisciplinary team which provided health information and psychosocial support. They were advised about the risk of vertical transmission though breastfeeding and formula milk was provided to safeguard the infants’ nutrition. Children of carrier mothers were monitored for at least three years after the birth on the paediatric infectology department of HUPE. There were two exceptions: one who was followed up at her local health care center and another who was lost to follow up.

### Screening and confirmation tests

Blood for HTLV-1/2 screening was collected either during the routine antenatal care or at the admission for delivery by chemiluminescent microparticle immunoassay (CMIA—Architect rHTLV-I/II, Abbott). Children’s samples were also screened by CMIA within the month after birth, at six months, one and two years of age. Infection was confirmed if the child remained seropositive after 24 months, and additional yearly exams were performed for follow-up. Reactive samples were confirmed by Western blot (WB, Inno Lia HTLV-I/II score Biomerieux). Two pregnant women with positive screening tests and negative WB results were considered false positive and allocated to the negative group in the statistical analysis. Routine antenatal screening for sexually transmitted infections (STI) in Brazil consists of VDRL test for syphilis and ELISA tests for HIV, hepatitis B and C. In case of positive screening the confirmation tests are FTA-Abs for syphilis and western blot for HIV. All tests were done at HUPE/UERJ’s Clinical Analysis Laboratory.

### Ethical aspects

This research complies with the Declaration of Helsinki and the Resolution 466 of December 12, 2012 of the Brazilian Ministry of Health. The project was approved by the Rio de Janeiro State University Research and Ethics Committee (COEP-UERJ, process 034.3.2012) and sponsored by the Fundação de Amparo à Pesquisa do Estado do Rio de Janeiro (FAPERJ, E-26/110.351/2012). Written informed consent was obtained from all the subjects and from the legally authorized representatives of the minors who agreed to take part in the research. Anonymity and data confidentiality were guaranteed.

### Data analysis

Means, medians, standard deviations and percentages were used to describe the results. Medians were used to define the cutoff point used to convert numerical variables to categorical ones (age, family income, number of partners and number of pregnancies). The following variables were considered as adverse pregnancy outcomes: fetal demise, perinatal death, maternal hypertensive syndrome, preterm birth and admission to the neonatal intensive care unit. A composite variable named ‘adverse outcome’ was created in view of the low frequency of each adverse obstetric outcome studied. Missing data were excluded from the statistical analyses. Mann-Whitney and Fisher’s exact tests were used to compare categorical variables between the HTLV-1/2 positive (G1) and negative (G2) groups. Multilevel logistic regression was performed to assess the factors associated to HTLV-1/2 infection. Age, number of pregnancies, coinfection with other STI, condom use in pregnancy and number of antenatal appointments were included in the model as fixed effects and "Hospital" was included as random effects (random intercept). The Epi-info software version 3.5.2 and the R-Project version 3.3.1 were used for building the database and performing the statistical analyses.

## Results

Prevalence of HTLV-1/2 infection in this study population was 0.74% (12/1628), with no significant difference between the hospitals (0.82% in HUPE, 0.67% in Mesquita; p = 0.78).

Among the sociodemographic characteristics (Table 1) only maternal age was significantly different between the HTLV positive and negative groups. The age was over 24 years in 83% (n = 10) of the HTLV+ group (G1) and in 53% of G2 (p = 0.03). Most women in both groups reported being non-white (p = 0.99); having at least 10 years of formal education (p = 0.99) and being in a stable marital relationship (p = 0.74). About half the patients in both groups had monthly household income higher than two minimum wages (p = 0.99). No subjects reported behavioural risk factors like the use of IV drugs, having multiple sexual partners and being a sex worker.

**Table 1:** Sociodemographic characteristics in HTLV-1/2 positive and negative patients.

		HTLV +	HTLV –	Odds Ratio (CI 95%)	P-value
		n (%)	n (%)		
**Age (years)**	> 24	10 (83%)	856 (53%)	4.44 (1.07–29.84)	0.03*
	≤ 24	2 (17%)	760 (47%)		
**Ethnicity**	White	9 (75%)	1,127 (70%)	0.78 (0.21–2.89)	0.49
	Non-white	3 (25%)	482 (30%)		
**Schooling (years)**	≥ 10	8 (67%)	1,069 (63%)	0.98 (0.29–3.28)	0.61
	< 10	4 (33%)	543 (37%)		
**Marital status**	Married	7 (58%)	1,071 (67%)	0.70 (0.22–2.22)	0.54
	Others	5 (42%)	537 (33%)		
**Household** **income (mw)**	< 2	6 (50%)	817 (51%)	0.98 (0.31–3.04)	0.97
	≥ 2	6 (50%)	799 (49%)		
**Place of birth**	RJ	10 (83%)	1,412 (87%)	1.38 (0.30–6.36)	0.46
	Others	2 (17%)	204 (13%)		

CI, confidence interval. mw, minimum wage.

* statistically significant.

Sexual and reproductive characteristics are summarized in Table 2. HTLV-1/2 positive women (G1) were more frequently coinfected with another STI than patients in G2 (41.7% vs. 8.9%, p< 0.01). Three of the HTLV-1/2 positive women had syphilis, one had HIV and another had histopathologically confirmed HPV condilomata. Most women in both groups had their first antenatal visit before 12 weeks of pregnancy (66.6% and 59.2%, p = 0.46). The number of antenatal appointments was higher in the HTLV-1/2 positive group, even after the multivariate analysis (81.8% vs. 52%, p = 0.03). Regarding the obstetric history, around 2/3 of infected women had more than two previous pregnancies (66.7%), while the proportion was inverse in G2 (34.9%). Number of sexual partners, previous reproductive history and frequency of condom use were similar in both groups.

**Table 2:** Sexual and reproductive characteristics of HTLV-1/2 positive and negative patients.

		HTLV +	HTLV -	Odds ratio (CI 95%)	P-value
		n (%)	n (%)		
**Sexual partners**	> 3	4 (50.0)	392 (33.2)	2.01 (0.50–8.10)	0.26
	≤ 3	4 (50.0)	790 (66.8)		
**STI coinfection**	Yes	5 (41.7)	144 (8.9)	0.13 (0.04–0.43)	< 0.01*
	No	7 (58.3)	1,472 (91.1)		
**Condom in pregnancy**	Yes	4 (33.3)	300 (20.0)	0.50 (0.15–1.67)	0.20
	No	8 (66.7)	1,203 (80.0)		
***Gravida***	> 2	8 (66.7)	555 (34.9)	3.72 (1.12–12.4)	0.02*
	≤ 2	4 (33.3)	1,034 (65.1)		
**Previous miscarriage**	Yes	3 (25.0)	355 (22.3)	0.86 (0.23–3.20)	0.52
	No	9 (75.0)	1,235 (77.7)		
**Previous adverse outcomes**	Yes	5 (41.7)	438 (27.5)	0.53 (0.17–1.69)	0.27
	No	7 (58.3)	1,152 (72.5)		
**Age at first intercourse**	≤ 16	8 (66.7)	765 (63.1)	0.85 (0.26–12.86)	0.53
	> 16	4 (33.3)	447 (36.9)		
**Antenatal appointments**	≥ 6	9 (81.8)	771 (52.0)	0.24 (0.05–1.12)	0.04*
	< 6	2 (18.2)	712 (48.0)		
**GA at first antenatal appointment**	1 Tri	6 (66.7)	805 (59.2)	0.73 (0.18–2.92)	0.46
	>1 Tri	3 (33.3)	554 (40.8)		

CI, confidence interval. STI, sexually transmitted infection. GA, gestational age.

* statistically significant.

The prevalence of comorbidities was high in the study population (37.6%), being significantly more frequent in HUPE (78.1%) than in HEM (4.8%, p<0.001). However, the rate was similar between the infected and non-infected groups (p = 0.16).

There were five adverse pregnancy outcomes in the HTLV positive group. The difference was not significant when compared to the negative group (p = 0.33) (Table 3). There were three cases of coinfection with syphilis (3/12) and three of premature rupture of membranes (PROM -3/12) at term. One patient with PROM also had syphilis, but the other two cases had no other risk factors (2/12; 16.6%). The prevalence of PROM in the HTLV-1/2 negative group was 4.7% (71/1,518). Although the difference seems significant, it was not confirmed by Fisher’s exact test (p = 0.26), probably due to the small number of cases. One HTLV-1/2-infected newborn was admitted to the neonatal intensive care unit (NICU) due to prolonged PROM. Among non-infected babies, 137 were admitted to the NICU (8.3% vs. 10.0%, p = 0.66). There were missing data on 210 seronegative pregnancy outcomes.

**Table 3:** Pregnancy outcomes in HTLV-1/2 positive and negative patients.

		HTLV +	HTLV -	Odds ratio	P-value
		n (%)	n (%)	(CI 95%)	
**Admission to the NICU**	Yes	1 (8.3)	137 (10.0)	1.23	0.66
	No	11 (91.7)	1,228 (90.0)	(0.16–9.58)	
**Miscarriage**	Yes	0 (0.0)	25 (1.6)	0	0.82
	No	12 (100.0)	1,581 (98.4)		
**Fetal demise**	Yes	1 (8.3)	43 (2.8)	0.32	0.30
	No	11 (91.7)	1,469 (97.2)	(0.04–2.55)	
**Hypertensive syndrome**	Yes	3 (25.0)	272 (18.3)	0.67	0.38
	No	9 (75.0)	1,217 (81.7)	(0.18–2.49)	
**Preterm birth**	Yes	0 (0.0)	112 (7.4)	0	0.40
	No	12 (100.0)	1,400 (92.6)		
**Any adverse outcome**	Yes	5 (41.7)	437 (28.8)	0.57	0.33
** **	No	7 (58.3)	1,081 (71.2)	(0.18–1.79)	

CI, confidence Interval. NICU, neonatal intensive care unit

* statistically significant

Missing data were excluded from the analysis

Differences in maternal age, STI coinfection and the number of antenatal visits remained significant after multivariate logistic regression (Table 4).

**Table 4:** Factors associated to increased prevalence of HTLV-1/2 infection.

	Odds ratio (CI 95%)	P-value
**Age**	1.11 (1.01–1.23)	0.037*
***Gravida***	2.19 (0.51–11.16)	0.310
**STI coinfection**	9.27 (2.32–36.54)	0.001*
**Condom in pregnancy**	0.86 (0.19–3.36)	0.838
**Antenatal visits**	5.31 (1.24–37.10)	0.043*

CI, confidence interval. STI, sexually transmitted infection.

* statistically significant.

Multilevel Logistic regression model–dependent variable: HLTV; fixed effects: age, number of pregnancies, coinfection with other STI, condom use in pregnancy, number of antenatal appointments; random effects: hospital.

Each additional year of maternal age increased the chance of being HTLV-1/2 positive in 11% (OR = 1.11). Having another STI increased 9 times the chance of being infected (OR = 9.27). In G1 there was a higher frequency of antenatal visits (OR = 5.31) (Table 4).

Among the children of the 12 HTLV-1/2 infected mothers, there was one fetal demise, one infant death and one loss of follow-up. The fetal death occurred at 24 weeks of pregnancy and the infant died at two months of age due to pneumonia. One child had its seroconversion (1/9) confirmed after two years of age. She was born at January 2013 and remains asymptomatic under medical surveillance at the HUPE to date. Her mother referred breastfeeding for less than one month. The other eight children were periodically monitored, seven at the pediatric infectology department of UERJ and one at its local health care center. Despite their economic difficulty, their mothers reported avoiding breastfeeding since they were aware of their carrier status.

## Discussion

The HTLV-1/2 prevalence found was consistent with the study previously published by our group which recruited only women admitted for delivery [24].

The sociodemographic profile of both groups was similar except for the older age found in the infected group. This is in accordance with the international literature on the disease’s epidemiology [4] and with the studies performed in Brazilian areas with high prevalence of HTLV-1/2 [17, 25,26]. On the other hand, in a large research performed in Gabon, where HTLV-1/2 prevalence is over 10%, there was no sociodemographic difference between the groups [11]. The multivariate analysis of sexual and reproductive characteristics found two significant differences between the infected and non-infected groups: STI coinfection and number of antenatal visits. The increased frequency of STI coinfection in HTLV-1/2 carriers, particularly syphilis, was consistent with studies from endemic areas [12,14,27,28], although it’s not a universal finding across publications [11,13,15,26,29]. On the Gabon research there were twice as many cases coinfected with syphilis than controls, however statistical significance was not reached, probably because insufficient sample size [11]. The latest Salvador study found that 21.5% of HTLV-1/2 infected subjects also had syphilis (OR = 36.7) [28]. As for the higher number of antenatal visits in the HTLV-1/2 positive group, it cannot be explained by the knowledge of the carrier status itself since only one patient was aware of the infection at the beginning of the pregnancy. There was also no significant correlation with the presence of comorbidities (p = 0.38). The hypothesis of the more frequent antenatal visits being due to these women having more previous adverse pregnancy outcomes seemed significant (p = 0.04), but its confidence interval was too wide (0.91–10.2). It is true, though, that the lack of significance could be caused by the small number of cases. A previous case-control study which addressed this variable also failed to find any difference between infected and non-infected patients [11].

Regarding the reproductive history, it’s striking that almost half of the carrier mothers (5/12) had previous adverse obstetric outcomes (three first trimester miscarriages, one fetal demise and one FGR with neonatal death). Three of these women had no comorbidities. It must be said however, that no causal link to HTLV-1/2 can be inferred since their infectious status was unknown during the previous pregnancies. The prevalence of early miscarriages was similar (*circa* 21%) among HTLV-1/2-positive and negative patients (p = 0.86). This was in accordance with data from other endemic areas [11,12].

Regarding the adverse pregnancy outcomes studied, no difference was observed between infected and non-infected patients. It’s important to stress that this finding cannot be generalized in view of the small sample size and the low incidence of the outcomes. The two studies reporting on obstetric results of HTLV-1/2 infected women did not find association between the infection and adverse pregnancy outcomes as well [11,12]. The Gabon research found a trend for preterm delivery and complicated pregnancies in HTLV-1/2 positive women [11]. Unfortunately, even this study, which followed 45 infected patients and 90 controls, was underpowered for this statistical analysis.

There were no cases of preterm delivery or low birth weight among HTLV-1/2 positive patients, in accordance with the findings of Bittencourt et al [12]. There was one case of fetal growth restriction and intrauterine demise in an otherwise healthy HTLV-1 infected mother. This woman reported two previous adverse pregnancy outcomes, but it’s unknown whether she was already infected at the time. At the Salvador case-control study[12], there were also fetal deaths on the HTLV-1/2 carrier mothers’ group, but those happened in patients with additional comorbidities such as hypertension and falciform anaemia. Additionally, that study reported three cases of hypertension in pregnancy and one admission to the NICU due to neonatal sepsis after PROM.

Among the infected mothers there were three cases of term PROM (25%). In a recent Brazilian study using data from the Ministry of Health [30], PROM was found to complicate approximately 4.2% of all livebirths in the country. This number is in line with the prevalence found in our HTLV-1/2 negative group. Uterine inflammation and sexually transmitted infections have been shown to be associated with obstetric complications such as PROM [31–34]. HTLV-1/2 infection is also known to be linked to different inflammatory and infectious manifestations. Thus, it seems reasonable to interrogate whether HTLV-1/2 infection may increase the risk of PROM. Unfortunately, the largest study on HTLV-1/2 pregnancy outcomes, performed in Gabon, didn’t assess the incidence of PROM [11]. This finding prompts the need for further research, adequately powered to elucidate the matter.

In our study, the mother of the only infected child reported breastfeeding for less than one month. Mother-to-child transmission of HTLV-1/2 occurs mainly through breastfeeding, ranging from 3.9% to 22% in endemic areas [35]. The policy of universal HTLV-1/2 antenatal screening and contraindication of breastfeeding for infected mothers at the Nagasaki province reduced the local MTC transmission rate from 20.3% to 2.5% [16], which are the known seroconversion rates of prolonged breastfeeding (> 6 months) and exclusive bottle-feeding, respectively. However, even with shorter periods of breastfeeding the MTC transmission rate is greater than using only infant formula (7.4% vs. 2.5%) [22]. Other possible reasons for the MTC transmission in this case are peripartum infection or additional risk factors such as: high maternal antigenemia, concentration of gp46 HTLV-1/2 antibodies, the presence of anti-Tax antibodies, or the human leucocyte antigen system (HLA) type concordance between mother and child [17,18,35,36,37,38,39]. The hypothesis of peripartum infection seems unlikely since the child was delivered by caesarean section due to hypertensive syndrome without PROM or labour. A limitation of this study is that it could not assess the other variables mentioned, such as proviral load and HLA type. Other two Brazilian studies report MTC transmission after less than a month of breastfeeding. In both cases the mothers’ proviral loads were extremely high [17, 37]. Another limitation of the study was the small sample of infected patients and the low frequency of adverse pregnancy outcomes, which were grouped for the statistical analysis. A study which is properly powered for statistical analysis on this matter would require a much greater sample size, and that may prove impeditive in areas of intermediate prevalence such as ours.

On the other hand, a strength of the study is that the children of infected mothers were followed up for three years, a gold standard set by the Nagazaki study group [16]. This was proposed since some cases of seropositivity in children are caused by maternal antibodies, which generally disappear after 12 months of life. Our MTC transmission rate was 1/9 (11%), similar to the ones found in Haiti and Guyana [11,35].

The study confirmed the high prevalence of HTLV-1/2 in pregnant women at the metropolitan area of Rio de Janeiro and found no sociodemographic difference between infected and non-infected patients. Carrier mothers frequently reported previous adverse pregnancy outcomes (5/12), but at the current pregnancy there was only one unexplained fetal demise (growth restricted) and one admission to the NICU due to sepsis. There was a significant association to other STI, but the intriguing point was the number of PROM cases among infected women (3/12).

Since there is no treatment or immunization for HTLV-1/2, preventive measures are currently the only effective way to break the chain of transmission. Thus, it is vital to increase awareness about the infection among health providers and the population. Safe sex campaigns are already extensive, therefore tackling MTC transmission is likely to have the most significant effect on the longitudinal perpetuation of the virus and the reduction of HTLV-1/2 associated diseases, particularly in endemic areas. There are no studies on antiretroviral therapy or mode of delivery to address the potential for reducing MTC transmission. Thus, avoidance of breastfeeding remains the only effective way to block the MTC transmission of the virus. The use of this strategy as a public policy in low income areas is still not a consensus, since breastfeeding also plays a role in reducing infant mortality and morbidity through immunity boosting and protecting against infections.

Since there is no clear difference in the sociodemographic profile of HTLV-1/2 carriers, routine prenatal screening at endemic areas is supported by several research groups [9,10,13,15–17,21,25,27,28,30,31,40–42]. Considering the number of livebirths at the metropolitan area of Rio de Janeiro (236,960 LB in 2016) and the HTLV-1/2 seroprevalence in pregnant women found in this study, the introduction of local routine antenatal screening could avoid over one thousand cases of MTC transmission in a year[41]. A similar estimate was found by a recent epidemiological study [42].

Further studies are needed on the cost-effectiveness of such strategy across different prevalence areas and socioeconomic resources. Moreover, epidemiological mapping of prevalence and mother-to-child transmission is needed to guide public health policies on antenatal screening and management. Additionally, better understanding of the burden of the infection in pregnancy may help to improve HTLV-1/2 infected mothers’ antenatal care and their children’s outcome.

## Supporting information

S1 Database HTLV PRENATAL. Patients database.
(XLS)

## References

[1] Poiesz BJ, Ruscetti FW, Gazdar AF, Bunn PA, Minna JD, Gallo RC. Detection and isolation of type C retrovirus particles from fresh and cultured lymphocytes of a patient with cutaneous T-cell lymphoma. Proc Natl Acad Sci USA. 1980; 77:7415–9. 10.1073/pnas.77.12.7415 6261256 PMC350514

[2] Gallo RC. History of the discoveries of the first human retroviruses: HTLV-1 and HTLV-2. Oncogene. 2005; 24(39):5926–30. 10.1038/sj.onc.1208980 16155599

[3] Mahieux R, Gessain A. The human HTLV-3 and HTLV-4 retroviruses: new members of the HTLV family. Pathol Biol. 2009; 57(2): 161–6. 10.1016/j.patbio.2008.02.015 18456423

[4] Gessain A, Cassar O. Epidemiological aspects and world distribution of HTLV-1 infection. Front Microbiol. 2012; 3:388 10.3389/fmicb.2012.00388 23162541 PMC3498738

[5] Ishak R, Vallinoto AC, Azevedo VN, Ishak M de O. Epidemiological aspects of retrovirus (HTLV) infection among Indian populations in the Amazon Region of Brazil. Cad Saude Publica. 2003; 19(4):901–14. 10.1590/S0102-311X2003000400013 12973556

[6] Paiva A, Casseb J. Origin and prevalence of human T-lymphotropic virus type 1 (HTLV-1) and type 2 (HTLV-2) among indigenous populations in the Americas. Rev Inst Med Trop. 2015; 57(1):1–13. 10.1590/S0036-46652015000100001 PMC4325517 25651320

[7] Roucoux DF, Murphy EL. The epidemiology and disease outcomes of human T-lymphotropic virus type II. AIDS Rev. 2004; 6(3):144–54. 15595431

[8] Guimarães de SV, Lobato Martins M, Carneiro-Proietti AB, Januário JN, Ladeira RV, Silva CMS, et al High prevalence of HTLV-1 and 2 viruses in pregnant women in São Luís, state of Maranhão, Brazil. Rev Soc Bras Med Trop. 2012; 45(2):159–62. 10.1590/S0037-86822012000200004. 22534984

[9] Carneiro-Proietti AB, Ribas JG, Catalan-Soares BC, Martins ML, Brito-Melo GE, Martins-Filho AO, et al Infection and disease caused by the human T cell lymphotropic viruses type I and II in Brazil. Rev Soc Bras Med Trop. 2002; 35: 499–508. 10.1590/S0037-86822002000500013. 12621671

[10] Gonçalves DU, Proietti FA, Ribas JG, Araújo MG, Pinheiro SR, Guedes AC, et al Epidemiology, treatment, and prevention of Human T-cell leukemia virus type1-associated diseases. Clin Microbiol Rev. 2010; 23(3):577–89. 10.1128/CMR.00063-09 20610824 PMC2901658

[11] Ville Y, Delaporte E, Peeters M, Leruez M, Glowaczower E, Fernandez H. Human T-cell lymphotropic virus type I infection and pregnancy: a case-control study and 12-month follow-up of 135 women and their infants. Am J Obstet Gynecol. 1991; 165(5 Pt 1): 1438–43. 1957877 10.1016/0002-9378(91)90387-7

[12] Bittencourt Al, Dourado I, Filho PB, Santos M, Valadão E, Alcantara LC, et al Human T-cell lymphotropic virus type I infection among pregnant women in northeastern Brazil. J Acquir Immune Defic Syndr. 2001; 26(5):490–4. 11391171 10.1097/00126334-200104150-00016

[13] Dal Fabbro MMFJ, Cunha RV, Bóia MN, Portela P, Botelho Ca, Freitas GMBF et al HTLV-1/2 infection: prenatal performance as a disease control strategy in State of Mato Grosso do Sul. Rev Soc Bras Med Trop. 2008; 41(2):148–51. 18545834 10.1590/s0037-86822008000200003

[14] Lima LH, Viana MC. Prevalence and risk factors for HIV, syphilis, hepatitis B, hepatitis C, and HTLV-I/II infection in low-income postpartum and pregnant women in Greater Metropolitan Vitória, Espírito Santo State, Brazil. Cad Saude Publica. 2009; 25(3): 668–76. 19300855 10.1590/s0102-311x2009000300021

[15] Sequeira CG, Tamegão-Lopes BP, Santos EJM, Ventura AMR, Moraes-Pinto MI, Succi RCM. Descriptive study of HTLV infection in a population of pregnant women from the state of Pará, Northern Brazil/Estudo descritivo da infecção pelo HTLV em uma população de gestantes do Estado do Pará, norte do Brazil. Rev Soc Bras Med Trop. 2012; 45(4):453–6. 10.1590/S0037-86822012005000007. 22836660

[16] Hino S. Establishment of the Milk-borne transmission as a key factor for the peculiar endemicity of human T-lymphotropic virus type 1 (HTLV-1): the ATL Prevention Program Nagasaki. Proc Jpn Acad Ser B Phys Biol Sci. 2011; 87(4): 152–66. 10.2183/pjab.87.152 21558754 PMC3149377

[17] Paiva AM, Assone T, Haziot MEJ, Smid J, Fonseca LAM, Luiz ODC, et al Risk factors associated with HTLV-1 vertical transmission in Brazil: longer breastfeeding, higher maternal proviral load and previous HTLV-1-infected offspring. Sci Rep. 2018; 8(1): 7742 10.1038/s41598-018-25939-y 29773807 PMC5958084

[18] Takezaki T, Tajima K, Ito M, Ito S, Kinoshita K, Tachibana K, et al Short term breast-feeding may reduce the risk of vertical transmission of HTLV-I. The Tsushima ATL study group. Leukemia. 1997; 11 (Suppl. 3):60–2. 9209298

[19] Fujino T, Nagata Y. HTLV-I transmission from mother to child. J Reprod Immunol. 2000; 47:197–206. 10924751 10.1016/s0165-0378(00)00054-1

[20] Takahashi K, Takezaki T, Oki T, Kawakami K, Yashiki S, Fujiyoshi T, et al Inhibitory effect of maternal antibody on mother-to-child transmission of human T-lymphotropic virus type 1: the mother-to-child Transmission Study Group. Int J Cancer. 1991; 49(5): 673–7. 10.1002/ijc.2910490508. 1937953

[21] Ureta-Vidal A, Angelin-Duclos C, Tortevoye P, Murphy E, Lepère JF, Buigues RP et al Mother-to-child transmission of human T-cell-leukemia/lymphoma virus type I: implication of high antiviral antibody titer and high proviral load in carrier mothers. Int J Cancer. 1999; 82(6): 832–6. 10446450 10.1002/(sici)1097-0215(19990909)82:6<832::aid-ijc11>3.0.co;2-p

[22] Hisada M, Maloney EM, Sawada T, Miley WJ, Palmer P, Hanchard B, et al Virus markers associated with vertical transmission of human T lymphotropic virus type 1 in Jamaica. Clin. Infect. Dis. 2002; 34(12):1551–7. 10.1086/340537 12032888

[23] Fujino T, Iwamoto I, Otsuka H, Ikeda T, Takesako S, Nagata Y et al Apoptosis in placentas from human T-lymphotropic virus type I-seropositive pregnant women: a possible defense mechanism against transmission from mother to fetus. Obstet Gynecol. 1999; 94:279–83. 10432143 10.1016/s0029-7844(99)00322-1

[24] Monteiro DL, Taquette SR, Sodré Barmpas DB, Rodrigues NC, Teixeira SA, Villela LH, et al Prevalence of HTLV-1/2 in pregnant women living in the metropolitan area of Rio de Janeiro. PLoS Negl Trop Dis. 2014; 8(9):e3146 10.1371/journal.pntd.0003146 25188386 PMC4154655

[25] Magalhães T, Mota-Miranda AC, Alcantara LC, Olavarria V, Galvão-Castro B, Rios-Grassi MF. Phylogenetic and molecular analysis of HTLV-1 isolates from a medium sized town in northern of Brazil: tracing a common origin of the virus from the most endemic city in the country. J Med Virol. 2008; 80(11): 2040–5. 10.1002/jmv.21278 18814252

[26] Mello MA, da Conceição AF, Sousa SM, Alcântara LC, Marin LJ, Raiol MRS et al HTLV-1 in pregnant women from the Southern Bahia, Brazil: a neglected condition despite the high prevalence. Virol J. 2014; 11:28 10.1186/1743-422X-11-28 24524416 PMC3974122

[27] Moxoto I, Boa-Sorte N, Nunes C, Mota A, Dumas A, Dourado I, et al Sociodemographic, epidemiological and behavioral profile of women infected with HTLV-1 in Salvador, Bahia, an endemic area for HTLV. Rev Soc Bras Med Trop. 2007; 40(1): 37–41. 10.1590/S0037-86822007000100007. 17486251

[28] Nunes D, Boa-Sorte N, Grassi MFR, Taylor GP, Teixeira MG, Barreto ML, et al HTLV-1 is predominantly sexually transmitted in Salvador, the city with the highest HTLV-1 prevalence in Brazil. PLoS ONE. 2017; 12(2): e0171303 10.1371/journal.pone.0171303 28158226 PMC5291389

[29] Olbrich NJ, Meira DA. Soroprevalence of HTLV-I/II, HIV, syphilis and toxoplasmosis among pregnant women seen at Botucatu- São Paulo—Brazil: risk factors for HTLV-I/II infection. Rev Soc Bras Med Trop. 2004; 37(1):28–32. 10.1590/S0037-86822004000100008. 15042179

[30] Leal MD, Esteves-Pereira AP, Nakamura-Pereira M, Torres JA, Theme-Filha M, Domingues RM, Dias MA, Moreira ME, Gama SG.Prevalence and risk factors related to preterm birth in Brazil. Reprod Health. 2016, 13(Suppl 3):127 10.1186/s12978-016-0230-0 27766978 PMC5073982

[31] Bastek JA, Weber AL, McShea MA, Ryan ME, Elovitz MA. Prenatal inflammation is associated with adverse neonatal outcomes. Am J Obstet Gynecol. 2014; 210(5):450.e1–10. 10.1016/j.ajog.2013.12.024 24361788

[32] Romero R, Espinoza J, Gonçalves LF, Kusanovic JP, Friel LA, Nien JK. Inflammation in preterm and term labour and delivery. Semin Fetal Neonatal Med. 2006; 11(5):317–26. 10.1016/j.siny.2006.05.001 16839830 PMC8315239

[33] Blas MM, Canchihuaman FA, Alva IE, Hawes SE. Pregnancy outcomes in women infected with Chlamydia trachomatis: a population-based cohort study in Washington State. Sex Transm Infect. 2007; 83(4): 314–8. 10.1136/sti.2006.022665 17344249 PMC2598687

[34] Reitter A, Stücker AU, Linde R, Königs C, Knecht G, Herrmann E, et al Pregnancy complications in HIV-positive women: 11-year data from the Frankfurt HIV Cohort. HIV Med. 2014; 15(9):525–36. 10.1111/hiv.12142 24602285

[35] Carneiro-Proietti AB, Amaranto-Damasio MS, Leal-Horiguchi CF, Bastos RHC, Seabra-Freitas G, Borowiak DR, et al Mother-to-child transmission of human T-cell lymphotropic viruses-1/2: what we know, and what are the gaps in understanding and preventing this route of infection. J Pediatr Infect Dis Soc. 2014; 3:S24–S29. 10.1093/jpids/piu070 PMC4164183 25232474

[36] Proietti FA, Carneiro-Proietti ABF, Catalan-Soares ABC, Murphy EL. Global epidemiology of HTLV-I infection and associated diseases. Oncogene. 2005; 24(39):6058–68. 10.1038/sj.onc.1208968 16155612

[37] Ribeiro MA, Martins ML, Teixeira C, Ladeira R, Oliveira MF, Januário JN, et al Blocking vertical transmission of human T cell lymphotropic virus type 1 and 2 through breastfeeding interruption. Pediatr Infect Dis J. 2012; 31(11):1139–43. 10.1097/INF.0b013e318263215e 22683674

[38] Percher F, Jeannin P, Martin-Latil S, Gessain A, Afonso PV, Vidy-Roche A, et al Mother-to-Child Transmission of HTLV-1: Epidemiological aspects, mechanisms and determinants of mother-to-child transmission. Viruses. 2016; 8(2):40 10.3390/v8020040 PMC4776195 26848683

[39] Takahashi K, Takezaki T, Oki T, Kawakami K, Yashiki S, Fujiyoshi T, et al (1991) Inhibitory effect of maternal antibody on Mother-to-Child transmission of human T-lymphotropic virus type I: the Mother-to-Child Transmission Study Group. Int J Cancer 49(5):673–7. 10.1002/ijc.2910490508 1937953

[40] Biggar RJ, Ng J, Kim N, Hisada M, Li HC, Cranston B, et al Human leukocyte antigen concordance and the transmission risk via breast-feeding of human T cell lymphotropic virus type I. J Infect Dis. 2006; 193(2):277–82. 10.1086/498910 16362892

[41] DATASUS. Departamento de informática do SUS. Serviço de Informação sobre Nascidos vivos (SINASC). Available from: http://www2.datasus.gov.br/DATASUS/index.php?area=0205. Acessed 14Jan2018.

[42] Rosadas C, Malik B, Taylor GP, Puccioni-Sohler M. Estimation of HTLV-1 vertical transmission cases in Brazil per annum. PLoS Negl Trop Dis. 2018; 12(11): e0006913 10.1371/journal.pntd.0006913 30418973 PMC6261628

